# Hydrogen regulates the aryl hydrocarbon receptor, improving bronchopulmonary dysplasia in neonatal rats and RLE-6TN cells exposed to hyperoxia

**DOI:** 10.3389/fped.2025.1662922

**Published:** 2025-11-17

**Authors:** Mulin Liang, Feifei Song, Jin Wang, Chunli Yang, Huixi Yin, Wei Zhou

**Affiliations:** 1 Department of Neonatal Intensive Care Unit, The Fifth Affiliated Hospital of Southern Medical University, Guangzhou, China; 2 Department of Neonatology, Guangzhou Women and Children’s Medical Center, Guangzhou Medical University, Guangzhou, China

**Keywords:** bronchopulmonary dysplasia, hydrogen, endoplasmic reticulum stress, AHR, CPEB4

## Abstract

**Introduction:**

This study investigates the role and underlying mechanism of hydrogen (H₂) in hyperoxia-induced bronchopulmonary dysplasia (BPD), aiming to provide a theoretical foundation for developing effective BPD treatment strategies.

**Methods:**

A hyperoxia-induced BPD rat model and a rat type II alveolar epithelial cell (RLE-6TN) injury model were established. H₂ was administered to assess its effects on BPD rats, while hydrogen-rich medium was used to treat RLE-6TN cells to evaluate cell viability. *In vivo* and *in vitro* experiments were conducted to explore the regulatory influence of H₂ on the aryl hydrocarbon receptor (AHR). Additionally, AHR knockdown and overexpression experiments were performed to determine the impact of AHR on cell viability.

**Results:**

H₂ treatment ameliorated lung tissue pathology in BPD rats, reduced cellular apoptosis, enhanced the expression of surfactant proteins SP-A and SP-B, and modulated AHR and its downstream effector CPEB4, thereby alleviating endoplasmic reticulum (ER) stress. *IN vitro*, hydrogen-rich medium mitigated RLE-6TN cell injury, promoted AHR nuclear translocation, and activated CPEB4 expression. AHR overexpression enhanced RLE-6TN cell viability and exhibited strong binding affinity to the CPEB4 promoter.

**Discussion:**

H₂ alleviates ER stress and reduces apoptosis by regulating AHR and its downstream molecule CPEB4, thereby mitigating hyperoxia-induced BPD. The protective mechanism of H₂ may be closely associated with the modulation of the AHR–CPEB4 signaling pathway and the attenuation of ER stress.

## 1 Introduction

Bronchopulmonary dysplasia (BPD) is a chronic lung disease characterized by impaired pulmonary vascular development, arrested alveolarization, and consequent decline in lung function. It remains the most prevalent complication among extremely preterm infants born during a critical window of lung development (1–4). The pathogenesis of BPD is fundamentally rooted in the inherent vulnerability of the premature lung. Preterm birth disrupts the normal progression of the saccular stage (24–38 gestational weeks in humans) and impedes the transition to the alveolar stage, during which most alveoli form through septation and microvascular maturation (5). This developmental interruption produces several key susceptibilities that predispose the preterm lung to BPD. (i) Structural Vulnerability: At birth, the preterm lung contains underdeveloped airspaces (saccules rather than mature alveoli), thickened septa, reduced surface area for gas exchange, and a fragile, immature capillary network (1–4). These structural deficits render the lung poorly equipped to adapt to extrauterine respiration or withstand additional insults. (ii) Immature Antioxidant Defenses: Premature infants exhibit markedly reduced levels of key antioxidant enzymes, including superoxide dismutase, catalase, and glutathione (6, 7). This deficiency leaves the developing lung highly susceptible to oxidative injury, even at oxygen concentrations that are safe for term infants. (iii) Heightened Susceptibility to Oxygen Toxicity and Inflammation: Life-saving interventions such as mechanical ventilation and supplemental oxygen, while essential, are major contributors to BPD pathogenesis (8). Hyperoxic exposure overwhelms the immature antioxidant capacity (9), leading to excess reactive oxygen species (ROS) generation. ROS accumulation damages alveolar epithelial type II (AT2) cells—crucial for surfactant synthesis and tissue repair—and endothelial cells, triggering intense pulmonary inflammation (10). The combined oxidative and inflammatory insult disrupts signaling pathways essential for septation and angiogenesis. (iv) Impaired Reparative Capacity: Developmental arrest, epithelial and endothelial injury, and persistent inflammation collectively compromise the lung's intrinsic repair potential (11–13). The resulting pathology includes arrested alveolarization (simplified, enlarged airspaces), dysmorphic vasculature, and disrupted epithelial–mesenchymal crosstalk necessary for alveolar formation. Thus, prematurity establishes a pathological substrate of structural immaturity and antioxidant deficiency. Superimposed hyperoxia and mechanical ventilation exacerbate this vulnerability (8, 9), driving the hallmark features of BPD—alveolar simplification, impaired vascularization, AT2 cell depletion, and chronic inflammation. This synergistic interplay between developmental immaturity and postnatal oxidative stress underlies the rationale for using the hyperoxia-induced neonatal rat model (born during the analogous saccular stage) to study BPD pathogenesis and therapeutics (14–16). Neonatal rats exposed to hyperoxia recapitulate the morphological and molecular hallmarks of human BPD, while reflecting genetic heterogeneity similar to that observed in preterm infants (17).

Current clinical strategies to reduce BPD risk—such as prenatal corticosteroids, caffeine, pulmonary surfactant therapy, and adjunctive medications—offer only modest benefits (18, 19). Consequently, there is a critical need for novel interventions that target the oxidative and inflammatory pathways driving disease progression. Hydrogen (H₂) has emerged as a promising therapeutic molecule with potent antioxidant, anti-inflammatory, and anti-apoptoticproperties (20–22). Multiple studies demonstrate its efficacy in respiratory disorders, including chronic obstructive pulmonary disease (COPD) and acute lung injury (ALI) (23). Recent findings indicate that H₂ inhalation attenuates placental inflammation and lipopolysaccharide (LPS)-induced BPD by inhibiting the TLR4–NF*κ*B–IL6/NLRP3 signaling pathway and reducing cytokine and chemokine production (6). However, the precise mechanisms by which H₂ mitigates hyperoxia-induced BPD remain to be elucidated.

The aryl hydrocarbon receptor (AHR), a ligand-activated transcription factor, has recently been recognized as a pivotal modulator of oxidative stress and inflammation in developing lungs exposed to hyperoxia. Shivanna et al. demonstrated that reduced AHR activation in neonatal Ahr^d^ mice aggravates hyperoxia-induced alveolar simplification and inflammation (24). Similarly, Rao et al. reported that AHR deficiency in neonatal rats increases alveolar epithelial apoptosis and vascular injury under hyperoxic conditions (25), underscoring the receptor's protective role. Mechanistically, Bhattacharya et al. showed that AHR activation by its endogenous ligand kynurenine upregulates antioxidant enzymes such as nuclear factor erythroid 2-related factor 2 (Nrf2) and heme oxygenase 1 (HO-1), thereby reducing oxidative stress and alveolar injury in a preterm lung model (26). Further, Kuiper-Makris et al. revealed that hyperoxia elevates intracellular oxidative stress and promotes epithelial–mesenchymal transition (EMT) through AHR-dependent signaling (15). Complementary *in vitro* studies demonstrate that omeprazole-induced AHR activation suppresses ROS production and monocyte chemotactic protein-1 (MCP-1) expression, mitigating hyperoxia-induced injury in human lung adenocarcinoma (H441) cells (27). Collectively, evidence from animal, cellular, and mechanistic studies establishes AHR as a central regulatory node in hyperoxia-induced lung injury. Its activation confers significant protection against oxidative and inflammatory damage, positioning AHR signaling as a promising therapeutic target in the prevention and treatment of BPD.

The endoplasmic reticulum (ER) is a central organelle responsible for protein folding, lipid synthesis, and calcium homeostasis. It maintains close functional interactions with other organelles and is essential for intracellular equilibrium. Disruptions caused by oxidative stress, hypoxia, or nutrient deprivation can induce ER stress and activate the unfolded protein response (UPR). In bronchopulmonary dysplasia (BPD), hyperoxia-driven oxidative stress destabilizes ER homeostasis, leading to the accumulation of misfolded proteins—such as surfactant proteins SP-A and SP-B—and consequent UPR activation (28, 29). ER stress is intricately linked to BPD pathogenesis. Hyperoxia impairs the folding and secretion of SP-A and SP-B, both of which are essential for alveolar stability. The downregulation of SP-B in particular has been associated with alveolar hypoplasia and respiratory failure in BPD (30). Activation of UPR sensors such as PERK and IRE1α further amplifies lung injury by promoting inflammation and apoptosis through CHOP-mediated pathways and the release of pro-inflammatory cytokines including IL-6 and TNF-α (31). Li et al. demonstrated that formononetin suppresses inflammation, ER stress, and apoptosis in bronchial epithelial cells via the AhR/CYP1A1 pathway. Lee et al. reported that AHR deficiency alleviates oxidative-stress-driven mesangial activation, macrophage infiltration, and extracellular matrix accumulation in diabetic nephropathy, while Guerrina et al. showed that AhR deficiency increases susceptibility to ER stress (32–34). In another study, Feng et al. found that hydrogen (H₂) inhalation improved pulmonary function in rats with chronic hypoxic pulmonary hypertension (CH-PH) by mitigating inflammation, reducing oxidative stress, and preserving AhR protein levels (35). Collectively, these findings suggest that H₂ may relieve ER stress by attenuating oxidative stress, thereby restoring SP-A/SP-B expression and regulating AHR signaling.

This study investigates the protective effects and underlying mechanisms of H₂ in hyperoxia-induced BPD, aiming to identify novel therapeutic strategies that enhance lung growth and repair.

## 2 Methods

### 2.1 Animal modeling and intervention

One-week-old Sprague-Dawley (SD) rats (male or female, 15 ± 3 g) were obtained from Guangdong Zhiyuan Biomedical Technology Co., Ltd. Animals were housed under standard conditions with *ad libitum* access to food and water and maintained on a 12-h light/dark cycle. Each lactating female rat nursed nine neonatal pups, with 12 pups assigned to each time point. All animal procedures adhered to the ARRIVE guidelines (27) and the U.K. Animals (Scientific Procedures) Act (1986) and were approved by the Laboratory Animal Ethics Committee of Lai'an Technology (Guangzhou) Co., Ltd. (Approval No. G2025039). To investigate dynamic pathological changes during alveolar development, three time points were selected: Day 3 (early injury—oxidative stress and apoptosis), Day 7 (alveolar arrest—impaired secondary septation), and Day 14 (established BPD—persistent structural and functional deficits). Neonatal rats and their surrogate mothers were continuously exposed to the designated gas mixtures from postnatal Day 0 until euthanasia on Day 3, 7, or 14. The rats were euthanized via carbon dioxide (CO₂) asphyxiation at a flow rate of 30% chamber volume displacement per minute for 5 min, followed by cervical dislocation to ensure death. The bronchopulmonary dysplasia (BPD) model was induced by exposing neonatal rats to 90% oxygen from birth (D_0_). We evaluated time-dependent effects of hyperoxia and hydrogen (H₂) by randomizing neonatal rats into four groups and assessing them at three postnatal ages (postnatal days 10, 14, and 21; hereafter D3, D7, and D14). At each time point, the groups were: (i) Control (Con): pups reared in standard cages breathing ambient air (21% O₂) without H₂; (ii) H₂: pups housed in a closed chamber breathing 21% O₂ with 2% H₂; (iii) BPD: pups exposed to 90% O₂ in a sealed chamber to induce BPD; and (iv) BPD + H₂: pups exposed to 90% O₂ with 2% H₂ in a closed chamber. Each group at each time point included three rats (*n* = 3), yielding a total *N* = 36 (4 groups × 3 time points × 3 rats). This design enabled dynamic evaluation of lung pathology, apoptosis, and surfactant-protein expression during hyperoxic exposure and H₂ intervention. Gas concentrations within exposure chambers were continuously monitored using a hydrogen detector (Model XP-3140, Japan) and an oxygen detector (Model CY-12C, China). Con group pups were maintained in the same indoor standard cages. Environmental conditions were held at 22 °C–26 °C and 60%–70% relative humidity. Chambers were opened daily at 09:00 for <1 h to rotate dams between high-oxygen and room-air litters to prevent maternal oxygen toxicity, replace bedding, and replenish water and chow. The H₂ supply was provided by a unit designed and manufactured by Suzhou Moor Gas Equipment Co., Ltd. (Suzhou, China), capable of delivering H₂ at a saturated concentration of 2%. All exposures were conducted in transparent sealed chambers (length × width × height: 32 × 22 × 14 cm^3^). Hydrogen concentration was monitored in real time using a combustible gas detector (Aegisafe, China). SD rats were euthanized via CO₂ inhalation on days 3, 7, and 14, with death confirmed by the absence of respiration, heartbeat, and reflexes. Lung tissues were collected immediately. The right lung was snap-frozen at −80 °C for biochemical analyses. Hematoxylin–eosin (HE), immunohistochemical (IHC), and TUNEL staining were performed to confirm BPD development and assess the therapeutic effects of hydrogen treatment.

### 2.2 ELISA

In the experiment involving hyperoxia in hrdrogen-treated BPD modle, the animals were categorized into four groups: the Control group (Con), hydrogen group (H2), BPD group (BPD) and BPD + hydrogen group (BPD + H2).On the 14th day, the SD rat were anesthezed and blood was collected. After centrifugation at 1,500 rpm for 10 min, serum samples were assayed for ALT (Abcam ab234579), AST (Abcam ab263883), Crea (Mybiosource MBS749827), and urea (Mybiosource MBS2600001) using ELISA kits.

### 2.3 HE staining

4% paraformaldehyde was gently instilled into the lungs through the airway under 20 cm H2O hydrostatic pressure. The lung tissues were embedded in paraffin and cut into 5 μm sections. The sections were stained using hematoxylin staining solution (ZLI-9610, zsbio, China) and water-soluble eosin staining solution (G1002, Servicebio, China), and observed under an optical microscope (NIKON ECLIPSE E100). Morphological analysis was carried out adopting a double - blind design. Sample numbering was accomplished by independent researchers, and the analysts were not privy to the information of the experimental groups. To perform objective and quantitative morphological analysis, in this study, systematic image acquisition and analysis were conducted on each lung tissue section under a 40× objective lens. To guarantee the representativeness and objectivity of the data, six independent fields of view were randomly selected within the non-overlapping and non-continuous regions of each slice. The physical area of each field of view is 0.1 mm^2^, thus the total analysis area of each slice should be at least 0.6 mm^2^. All image analyses were carried out using the Image J software. To uphold the consistency of the analysis conditions, a uniform threshold range was set to precisely distinguish the target structure from the background, and all measurement tasks were completed by a researcher unaware of the experimental grouping information, aiming to minimize subjective deviation. Among these, the calculation of the integrated optical density (IOD) value is employed for the semi - quantitative analysis of immunohistochemical staining, and it is also based on the aforementioned standardized field of view. The radial alveolar count (RAC) and surface Area (SA) were assessed as previously described (36). RAC defined as the number of closed alveoli intersected by a line drawn perpendicularly from the terminal bronchiole to the nearest pleura. Septal thickness measurements were obtained from HE-stained sections. Alveolarization levels were quantified through mean cord length (Lm) and alveolar surface area. Lm, representing the distance between airspace walls. After adjusting for a shrinkage rate of 40%, the SA was computed using the equation SA = 4 × Tissue volume density (VDT) × lung volume/Lm × (1–0.4).

### 2.4 TUNEL staining

TUNEL staining kit (GDP1042, Servicebio, China) was used to detect the number of cell death in lung tissue, and the experiment was performed according to the instructions of the kit. Images were captured using a Minmei microscopy digital imaging system (mshot, China). The apoptosis rate was represented by the ratio of the area of TUNEL-positive cells to the total area of DAPI-stained nuclei. The average value of three sections per lung tissue was calculated, with three lung tissues per group.

### 2.5 Cell culture and processing

Rat type II alveolar epithelial cells, RLE-6TN (CRL-2300, ATCC), were conducted using certified materials obtained from ATCC (https://www.atcc.org/). RLE-6TN cells were seeded and cultured in Ham's F-12K medium (21127022, Gibco, USA), supplemented with 10% fetal bovine serum (FBS) (A5670701, Gibco, USA) and 1% penicillin-streptomycin solution (100 μg/ml) (15070063, Gibco, USA). The cells were maintained in a 37 °C incubator with 5% CO2. Subsequently, the cells were randomly assigned to one of four groups: the Control group (Con), hydrogen group (HRM), high oxygen group (Hyperoxia), and high oxygen + hydrogen group (Hyperoxia + HRM). The hydrogen-rich medium (HRM) was prepared following a previously described method38. H2 was dissolved in Ham's F-12K medium at a pressure of 0.4 MPa for 2 h to achieve supersaturation, resulting in a constant hydrogen concentration of 0.6 mm. The hydrogen concentration in the complete culture medium was measured using the methylene blue–redox titration method. A 5 ml sample of the hydrogen-containing culture medium was titrated with the methylene blue colloidal platinum (MB-Pt) reagent until the blue color just ceased to disappear, indicating the endpoint of the titration. Theoretically, the addition of one drop of the colloidal platinum MB-Pt reagent corresponds to a hydrogen concentration of 0.1 mmol/L. For instance, if 8 drops of the reagent are added, the resulting concentration of hydrogen that does not turn blue is 0.8 mmol/L. Following this pattern, if 12 drops are added, the concentration of hydrogen that does not turn blue would be 1.2 mmol/L. This process continues until the hydrogen-containing medium just turns blue. To determine the concentration of hydrogen gas in the hydrogen-containing solution, calculate the number of drops of colloidal platinum MB-Pt reagent added, divided by 10.

For cells requiring high oxygen treatment, a mixture of 5% CO2% and 95% O2 was introduced into the oxygen chamber, which was then placed in the incubator. In the protein extraction experiments from distinct cellular compartments, the RLE-6TN cells from diverse groups (Con, HRM, Hyperoxia, Hyperoxia + HRM) were rinsed twice with pre-chilled phosphate-buffered saline (PBS) and collected via nuclear and cytoplasmic proteins were extracted using the Nuclear and Cytoplasmic Protein Extraction Kit (P0027, Beyotime, China). The cells were washed with PBS, and cell pellets were collected by centrifugation. For cytoplasmic protein extraction, an appropriate volume of cytoplasmic extraction reagent was added to the pellets; the mixture was vortexed thoroughly to ensure complete cell lysis, followed by centrifugation. After centrifugation, the supernatant (containing cytoplasmic proteins) was immediately transferred to a pre-cooled tube. For nuclear protein extraction, the residual supernatant from the aforementioned centrifugation step was first thoroughly aspirated to avoid cytoplasmic contamination. An appropriate volume of nuclear extraction reagent was then added to the remaining pellet, and the mixture was vortexed vigorously at intervals to fully disperse the pellet. Following subsequent centrifugation, the supernatant (containing nuclear proteins) was immediately transferred to a fresh pre-cooled tube.

In the experiment involving the AHR ligand FICZ in hyperoxia-treated RLE-6TN cells, the cells were categorized into four groups: the Control group (Con), the FICZ group (FICZ), the Hyperoxia group (Hyperoxia), and the Hyperoxia + FICZ group (Hyperoxia + FICZ). The hyperoxia treatment was conducted as previously described, and the concentration of FICZ (SML1489, Sigma, USA) used was 10 μM.

### 2.6 Flow cytometry

For flow cytometry analysis of SP-A and SP-B, lung tissues were inflated via the trachea with dispase and kept in dispase and Collagenase Type IV at 37 °C for 40 min with frequent agitation. To achieve single cell suspensions, digested tissue was passed serially through 100-, 70- and 40-μm cell strainers. Cells were resuspended in FACS buffer, with a concentration adjusted to 5 × 10^6^ cells/ml. To isolate AT2 cells, single-cell suspensions from lung tissues were subjected to fluorescence-activated cell sorting (FACS) based on the following phenotypic profile: PI- (propidium iodide negative, to exclude dead cells), CD45-(negative for the pan-hematopoietic marker CD45, to exclude immune cells), CD31-(negative for the endothelial marker CD31, to exclude endothelial cells), EpCAM + (positive for the epithelial cell adhesion molecule EpCAM, to enrich epithelial cells), and LysoTracker + (positive for LysoTracker, a fluorescent dye that accumulates in the lamellar bodies characteristic of AT2 cells). RLE-6TN cells were fixed with 5% paraformaldehyde at room temperature for 30 min and subsequently blocked with 10% goat serum for 1 h at room temperature. The corresponding primary antibodies were then added and incubated at 4 °C overnight. Following this, the appropriate secondary antibodies were added at room temperature and incubated for an additional 30 min before detection using a flow cytometer (Accuri™, BD Biosciences, USA). Each sample documents 10,000 events for the purpose of analysis. The antibodies utilized in this study included: SP-A (1:500, 11850-1-AP, Proteintech, China), SP-B (1:500, 1034R, Yajimall, China), Goat Anti-Rabbit IgG H&L (Alexa Fluor® 488) (ab150077, Abcam, UK), and Goat Anti-Mouse IgG H&L (Alexa Fluor® 488) (ab150113, Abcam, UK). The rate of cell apoptosis was assessed using a flow cytometer in accordance with the instructions provided in the ANNEXIN V-FITC/PI Apoptosis Detection Kit (G1511, Solarbio, China).

### 2.7 IHC staining

Paraffin sections of lung tissue were deparaffinized and subsequently incubated in 3% hydrogen peroxide for 10 min. Following this, the sections were blocked with 10% goat serum at room temperature for 10 min and then incubated with primary antibodies at 37 °C for 2 h. Afterward, they were incubated with the corresponding secondary antibody at 37 °C for 30 min. The sections were rinsed three times with PBS and incubated in a horseradish peroxidase-labeled streptavidin working solution at 37 °C for 5 min. Color development was achieved using DAB chromogen for 10 min. The sections were then washed, sealed with resin, and photographed using the Mingmei Microscope Digital Imaging System (mshot, China). The expression levels of p-AHR, AHR, and CPEB4 were assessed using integrated optical density (IOD) to quantify staining intensity. Morphological analysis was conducted on each lung section with an area of at least 0.6 mm^2^ (six non - overlapping fields of view were randomly selected under a 40× objective lens, with each field of view having an area of 0.1 mm^2^). Image J software was employed to calculate the integrated optical density (IOD) values, with a consistent threshold setting. The IOD values, representing the total optical density of the stained regions, were calculated using ImageJ analysis software, which provided a measurement of antigen expression levels in the samples. The antibodies utilized in this study were as follows: p-AHR (1:1000, PA5-104880, Thermo Fisher, USA), AHR (1:800, 67785-1-IG, Proteintech, China), CPEB4 (1:250, 25342-1-AP, Proteintech, China), Goat Anti-Rabbit IgG H&L (HRP) (1:1000,GB23303, Servicebio, 1:200), and Goat Anti-Mouse IgG/HRP (1:1000, GB23301, Servicebio, 1:200).

### 2.8 Western blot (WB) analysis

Nuclear and cytoplasmic proteins cytoplasmic proteins were extracted using a Nuclear and Cytoplasmic Protein Extraction Kit (P0027, Beyotime, China). Total proteins from tissues and cells were extracted with pre-cooled RIPA lysis buffer (strong formula, Beyotime, #P0013B). For inhibition of protein degradation and dephosphorylation, 1 mm PMSF (Beyotime, #ST506) and a cocktail of protease and phosphatase inhibitors (Roche, #04693132001 & #04906837001) were added to the lysis buffers. After lysis, tissue samples were centrifuged at 12,000 rpm at 4 °C for 15 min, and the resulting supernatant was collected as total protein extract. Protein concentrations were accurately determined using a BCA protein quantification kit (Beyotime, #P0012).

To ensure the reliability and comparability of Western blot results, the total protein loading amount per well was standardized after calculation: 30 μg for tissue-derived samples and 20 μg for cell-derived samples. This loading volume was validated through preliminary experiments to confirm that the signal intensity of target bands remained within the linear range without oversaturation. All samples were mixed with 5× SDS-PAGE loading buffer and boiled at 100 °C for 10 min to achieve complete protein denaturation prior to electrophoresis. Following electrophoresis, proteins were transferred onto PVDF membranes, which were then blocked with 5% BSA for 1 h. Membranes were incubated with the appropriate primary antibodies overnight at 4 °C, followed by incubation with corresponding secondary antibodies at room temperature for 2 h. Protein bands were visualized using enhanced chemiluminescence (ECL) detection, and gray values were quantified using an automatic gel imaging analyzer.

The antibodies used in this study included: p-AHR (1:1000, PA5-104880, Thermo Fisher, USA), AHR (1:1000, MA1-513, Thermo Fisher, USA), CPEB4 (1:1000, PA5-25538, Thermo Fisher, USA), p-IRE1α (1:1000, PA5-105424, Thermo Fisher, USA), IRE1α (1:500, MA5-14991, Thermo Fisher, USA), GAPDH (1:10000, ab8245, Abcam, UK), β-actin (1:10000, ab8226, Abcam, UK), histone (1:10000, ab1791, Abcam, UK), Goat Anti-Rabbit IgG H&L HRP (1:10000, ab6721, Abcam, UK), and Goat Anti-Mouse IgG/HRP (1:5000, SE131, Solarbio, China). Band intensities of p-AHR were normalized to AHR using ImageJ software, while all other protein band intensities were normalized to GAPDH, also using ImageJ software.

### 2.9 AHR knockdown and overexpression

The si-NC, si-AHR, vector and the oe-AHR sequence were synthesized by Tsingke Biotech Co., Ltd. (Beijing, China). Following the manufacturer's guidelines, Lipofectamine 3000 (Invitrogen, United States) was employed to transfer small interfering RNAs. The cells were harvested 48 h after transfection. Detailed sequences are available in the Supplementary Material Data 1–3.

### 2.10 Cell viability assessment

Cell viability was assessed using the M5 HiPer Cell Counting Kit (MF128-01, Mei5bio, China). Cells were seeded in a 96-well plate at a density of 3 × 10^3^ cells per well. At 24, 48, and 72 h post-seeding, 10 μl of CCK-8 reagent was added to each well and incubated at 37 °C in the dark for 2 h. Subsequently, the absorbance at 450 nm was measured using a microplate reader (ELX800, BioTek, USA).

### 2.11 Quantitative real-time polymerase chain reaction (qPCR)

Cells were lysed using Trizol solution, and RNA was extracted with chloroform and isopropanol. The mRNA was reverse transcribed following the protocol provided by the M5 HiPer First Strand cDNA Synthesis Kit (MF011-01, Mei5bio, China), and the resulting cDNA was utilized for qPCR. The experimental procedures were conducted according to the PerfectStart® Green qPCR SuperMix Kit (MF013, Mei5bio, China). The primer sequences employed were: AHR (CACAGAGACCGGCTGAACAC, TGCTGAAAGCCCAGGTAATCT); CPEB4 (ACGGGTTTGGAGTGCTAGTG, CCCCTGGATTTTCTTCGGCT); and GAPDH (AATGACCCCTTCATTGAC, TCCACGACGTACTCAGCGC).

### 2.12 ChIP-quantitative polymerase chain reaction (ChIP-qPCR)

ChIP-qPCR was performed according to the instructions provided in the ChIP Assay Kit (ab156907, Abcam, UK). Cells were cross-linked using 37% formaldehyde, followed by treatment with 125 mm glycine. After lysis and sonication, the digested chromatin was mixed with antibodies and incubated overnight at 4 °C with shaking. Subsequently, magnetic beads were added to the immunoprecipitation reaction and mixed with shaking for 1 h at 4 °C. The immunoprecipitated chromatin DNA was then eluted and quantified using qPCR. The antibodies used included anti-AhR (ab2769, Abcam, UK) and an IgG isotype control (3900, Cell Signaling Technology, USA). Detailed primer sequences are provided in the Supplementary Material Data 4–5.

### 2.13 Immunofluorescence staining

RLE-6TN cells were fixed with 4% paraformaldehyde for 30 min. The cells were permeabilized with 0.5% Triton (9002-93-1, Solarbio, China) for 10 min and blocked with serum at 26 °C for 1 h. The cells were then incubated with CPEB4 antibody (1:250, 25342-1-AP, Proteintech, China) and AHR antibody (1:800, 67785-1-IG, Proteintech, China) at 4 °C overnight, followed with fluorescent secondary antibody, and incubated for 2 h at 26 °C. DAPI was used to stain the nuclei. Fluorescence intensity was observed using a laser confocal microscope (LSM 900, ZEISS, Germany). Quantify immunopositive cells using Image J software.

### 2.14 Statistical analysis

All experiments were performed in triplicate using independent biological replicates, and data are expressed as mean ± standard deviation. Data normality was verified with the Shapiro–Wilk test, and homogeneity of variances was assessed using Levene's test. For multiple group comparisons, one-way analysis of variance was conducted, followed by least significant difference *post hoc* testing when overall significance was observed (*P* < 0.05). Outliers were identified using the interquartile range (IQR) method, where values below Q₁ – 1.5 *×* IQR or above Q₃ + 1.5 *×* IQR were excluded. Statistical analyses were performed using GraphPad Prism 9.0 (GraphPad Software, La Jolla, CA, USA). A *P*-value <0.05 was considered statistically significant.

## 3 Results

### 3.1 H_2_ improves hyperoxia-induced BPD

**Figure 1:**
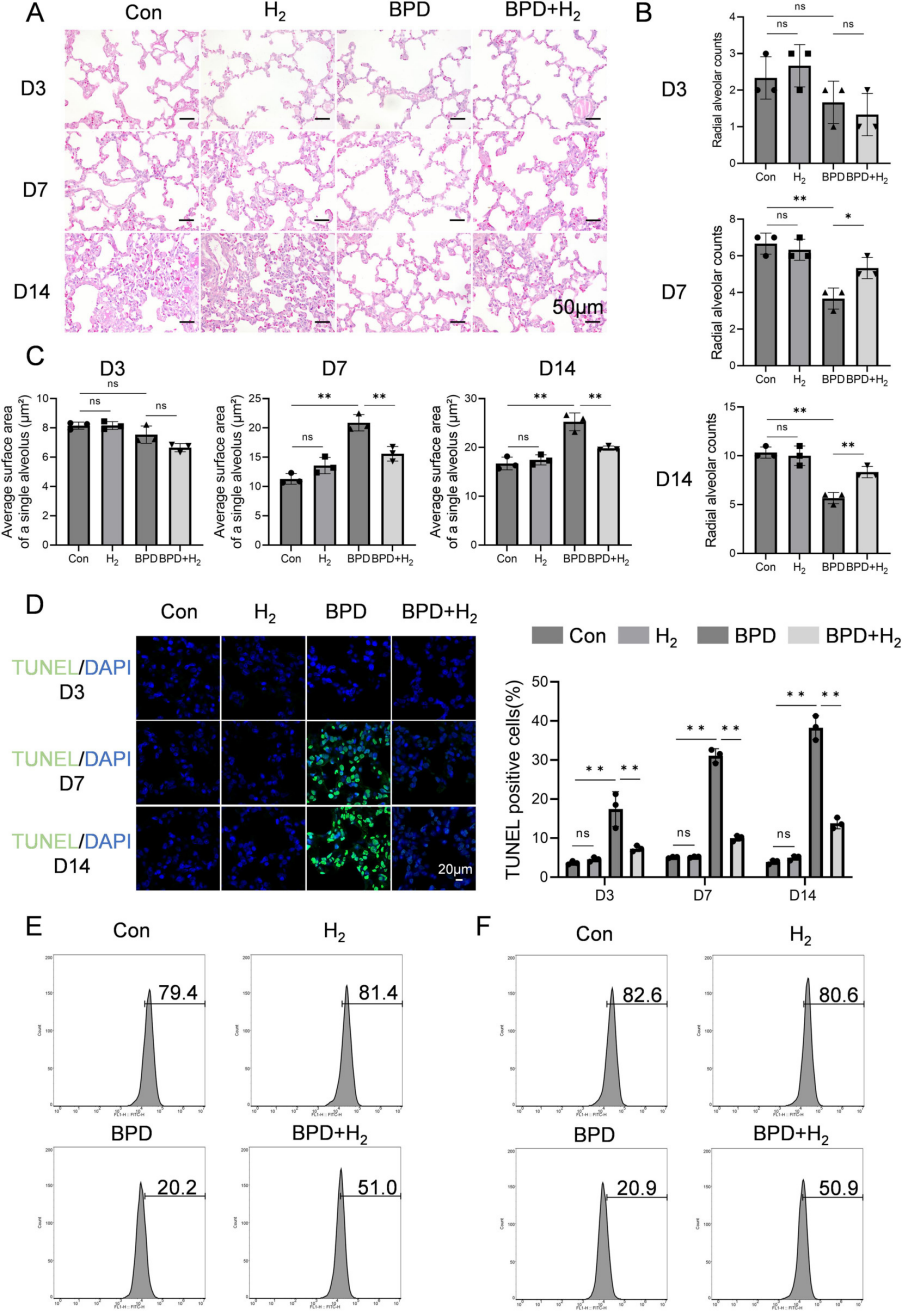
H_2_ ameliorates BPD induced by hyperoxia. **(A)** Hematoxylin–eosin staining was performed to assess the pathological conditions of the lung tissue in BPD rats, 20×, scale bar = 50 μm. **(B)** Radial alveolar count (RAC) analysis, AHR activation (phosphorylated form) is mainly enriched in the alveolar epithelial cell nuclei, while CPEB4 is highly expressed in the cytoplasm and perinuclear regions. **(C)** Quantification of the average surface area of a single alveolus (µm^2^). **(D)** TUNEL staining was performed to detect apoptosis in the lung tissue. **(E)** Flow cytometry was employed to measure the expression of SP-A in BPD rats on day 14 from the Con, H_2_, BPD, and BPD + H_2_ groups. **(F)** Flow cytometry was also used to evaluate the expression of SP-B on day 14 from the Con, H_2_, BPD, and BPD + H_2_ groups. Data are presented as mean ± SD. (*n* = 3).

The pathological alterations in rat lung tissue were evaluated using hematoxylin–eosin (HE) staining. As shown in Figures 1A–C, at Day 3 (early pathological stage), the BPD group exhibited mild thickening of alveolar septa, reduced density of alveolar branches, and a slight decrease in the radial alveolar count (RAC). However, these changes were not statistically significant. The total alveolar surface area—calculated as the product of alveolar number and mean individual alveolar surface area—remained unchanged. The minor reduction in RAC implied a compensatory increase in the mean linear intercept (Lm), reflecting the onset of alveolar developmental impairment. At Day 7 (progressive injury stage), alveoli in the control group displayed continued maturation, with an increased number of alveoli consistent with normal development. In contrast, the BPD group showed a significant reduction in alveolar number, enlargement of alveolar spaces, a marked decrease in RAC (*P* < 0.05), and a concurrent increase in Lm—hallmarks of delayed alveolarization. The average individual alveolar surface area was also significantly greater (*P* < 0.05). At this stage, the reduction in alveolar number outweighed the compensatory effect of alveolar enlargement. Together with septal thickening and structural disorganization, these changes resulted in a significant decline in total alveolar surface area, indicating alveolar simplification. By Day 14, alveoli in the control group appeared compact and uniform, whereas those in the BPD group were grossly enlarged, with significantly reduced RAC (*P* < 0.05), increased Lm, and elevated average alveolar surface area (*P* < 0.05), accompanied by a further decline in total alveolar surface area—confirming structural simplification. In comparison, the H₂-treated group exhibited a marked attenuation of these pathological features (*P* < 0.05), with alveolar morphology resembling that of the control group, restored RAC (counteracting BPD-induced reduction), and decreased average alveolar surface area.

As shown in Figure 1D, apoptosis in alveolar epithelial cells of BPD model rats was assessed using TUNEL staining, and the proportion of TUNEL-positive cells was quantitatively analyzed to reflect apoptotic intensity. Over time (Day 3 → Day 7 → Day 14), apoptosis in the BPD group followed a pattern of initiation → peak → persistence, with Day 7 representing the apoptotic apex—corresponding to the most pronounced mid-stage structural damage observed in HE staining. At Day 3, minimal green fluorescence was observed in the control group, indicating normal developmental status. In contrast, the BPD group displayed a slight increase in fluorescence, suggesting the onset of apoptosis and injury-induced cell death. The BPD + H₂ group exhibited intermediate fluorescence intensity, indicating that early H₂ intervention partially suppressed apoptosis. At Day 7, intense green fluorescence was evident in the BPD group, indicating extensive apoptosis. Fluorescence in the BPD + H₂ group was markedly lower (*P* < 0.05) and approached control levels, demonstrating that H₂ effectively inhibited cell apoptosis. By Day 14, persistent green fluorescence in the BPD group indicated ongoing cell death and incomplete repair, whereas the BPD + H₂ group showed substantially reduced fluorescence, suggesting sustained anti-apoptotic and cytoprotective effects of hydrogen.

The expression of surfactant proteins SP-A and SP-B was examined by flow cytometry. Compared with the BPD model group, the H₂-treated group exhibited significantly higher proportions of alveolar type II (AT2) cells expressing SP-A and SP-B (*P* < 0.05; Figures 1E,F). These findings indicate that hydrogen therapy preserves AT2 cell integrity under hyperoxic stress, maintaining their functional capacity for surfactant protein synthesis. Serum biochemical indices—alanine aminotransferase (ALT), aspartate aminotransferase (AST), creatinine (CREA), and urea (UREA)—were also evaluated across all groups (Control, H₂, BPD, and BPD + H₂). No statistically significant differences were observed among groups (*P* > 0.05; Supplementary Figure S1), indicating that hydrogen treatment did not induce systemic toxicity.

### 3.2 H_2_ regulates the AHR and CPEB4, alleviating ER stress

**Figure 2:**
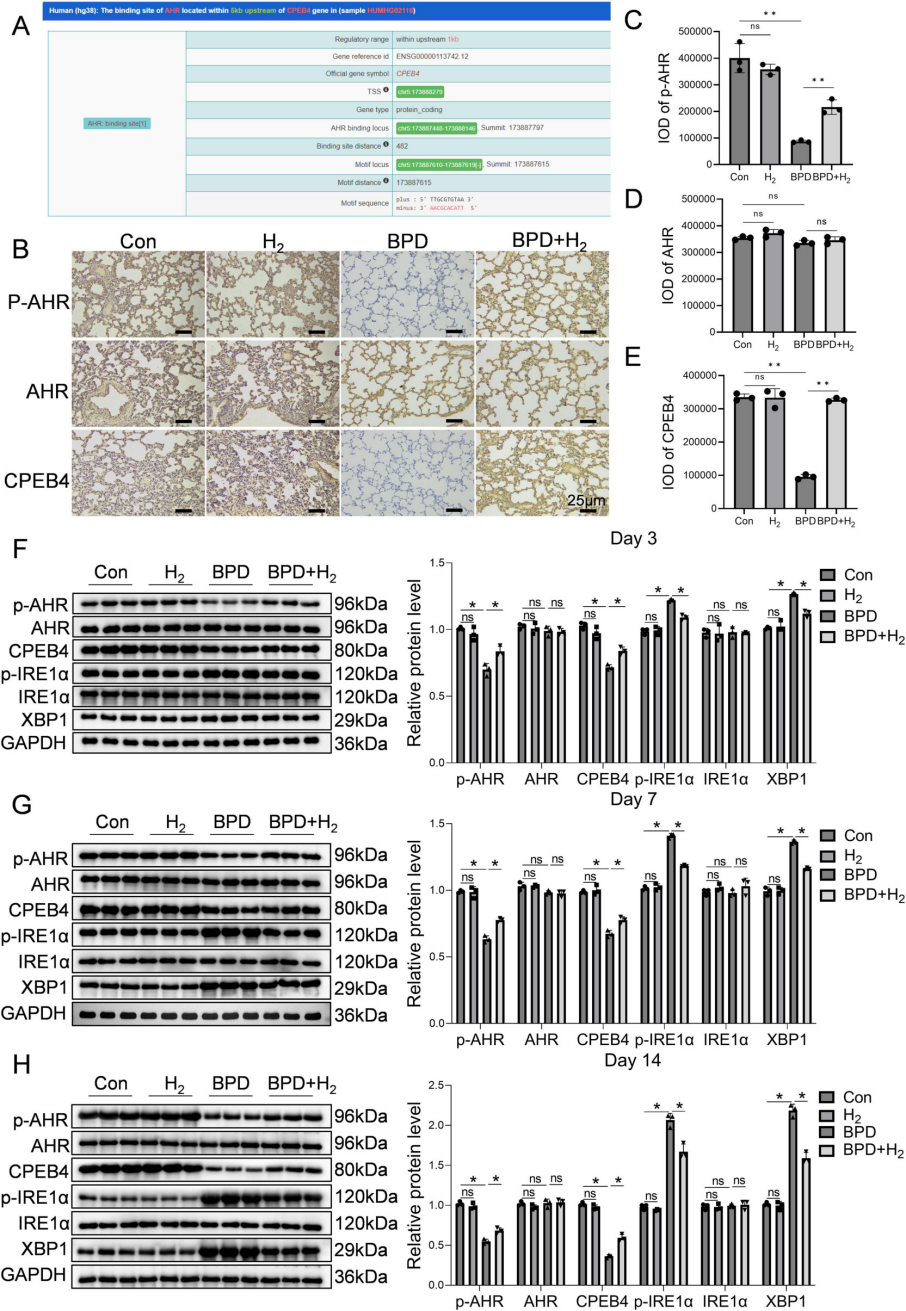
H_2_ activates AHR and its downstream molecule CPEB4, thereby reducing endoplasmic reticulum stress. **(A)** Predicted downstream regulators of AHR are presented. **(B)** Immunohistochemical detection of AHR activation and CPEB4 expression in the lung tissue is shown, 60×, scale bar = 25 µm, AHR activation (phosphorylated form) is mainly enriched in the alveolar epithelial cell nuclei, while CPEB4 is highly expressed in the cytoplasm and perinuclear regions. **(C–E)** IOD quantitative analysis of the protein expression of p-AHR, AHR, and CPEB4. **(F–H)** Protein expression of p-AHR, AHR, CPEB4, p-IRE1α, IRE1α, and XBP1 was assessed by western blotting on days 3, 7, and 14, and the protein bands were quantified. Data are presented as mean ± SD. (*n* = 3), *P < 0.05.

Bioinformatic prediction using ChIP-seq data (ENCODE database) revealed that aryl hydrocarbon receptor (AHR) directly binds to the CPEB4 promoter region, located 482 bp upstream of the TSS (Figure 2A). Immunohistochemistry for p-AHR, total AHR, and CPEB4 demonstrated specific nuclear localization of AHR/p-AHR and cytoplasmic localization of CPEB4 within alveolar epithelial and mesenchymal cells, consistent with expected subcellular distribution (Figure 2B). Quantitative analysis of staining intensity using integral optical density (IOD) revealed that p-AHR IOD was significantly lower in the BPD group than in controls (*P* < 0.01; Figure 2C), whereas total AHR showed no significant difference (Figure 2D). Similarly, CPEB4 IOD was significantly reduced in the BPD group (*P* < 0.01; Figure 2E). Notably, both p-AHR and CPEB4 IOD values were markedly increased in the BPD + H₂ group relative to the BPD group (*P* < 0.01*), indicating restoration by hydrogen therapy.

Western blot analysis corroborated these findings (Figures 2F–H). The p-AHR level was significantly reduced in the BPD group (*P* < 0.05) but recovered toward control levels in the BPD + H₂ group, without changes in total AHR. Conversely, phosphorylated IRE1α (p-IRE1α) was elevated in the BPD group (*P* < 0.05), decreased in the BPD + H₂ group, and returned to control levels, while total IRE1α remained unchanged, indicating hyperactivation of the IRE1α pathway in BPD. Similarly, CPEB4 expression was significantly decreased in the BPD group (*P* < 0.05) and restored by H₂ treatment. The XBP1 level, elevated in the BPD group (*P* < 0.05), was suppressed by H₂. Collectively, these results indicate that hydrogen restores CPEB4 expression, inhibits excessive p-IRE1α activation, and attenuates downstream XBP1 signaling, thereby mitigating ER stress–associated injury in BPD.

### 3.3 H_2_ reduces the damage to RLE-6TN cells

**Figure 3:**
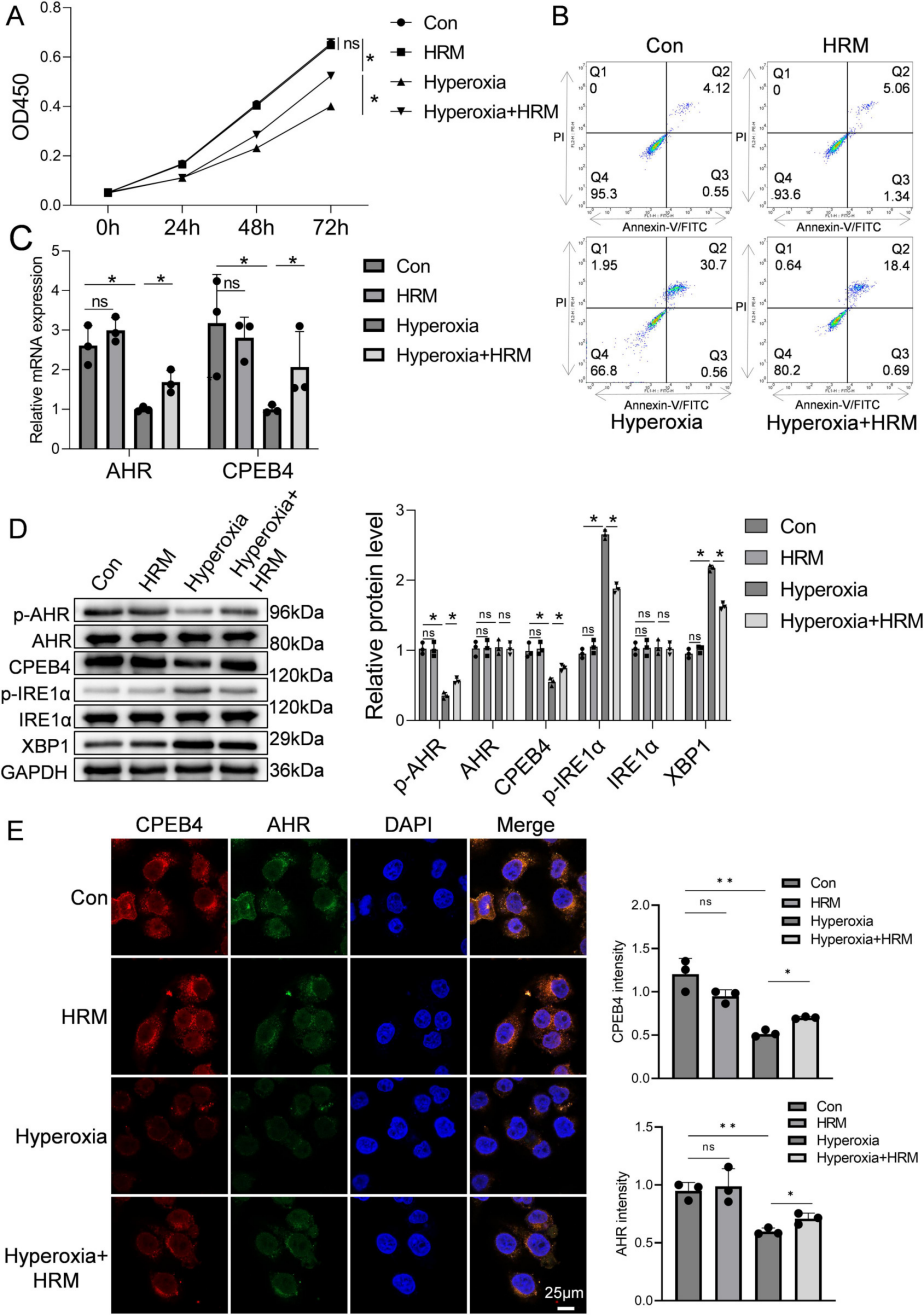
Effect of H_2_ on RLE-6TN cells in a hyperoxic environment was investigated. **(A)** CCK-8 assay was performed to assess the viability of RLE-6TN cells. **(B)** Flow cytometry was performed to further evaluate the apoptosis of RLE-6TN cells. **(C)** qPCR was conducted to measure the mRNA levels of AHR and CPEB4 in RLE-6TN cells. **(D)** Western blotting was performed to analyze the expression of p-AHR, AHR, CPEB4, p-IRE1α, IRE1α, and XBP1 in RLE-6TN cells, followed by quantitative analysis of the protein bands. **(E)** Immunofluorescence microscopy was used to observe the nuclear translocation of AHR in RLE-6TN cells, 60×, scale bar = 25 µm. Data are presented as mean ± SD. (*n* = 3), *P < 0.05.

The CCK-8 assay was used to assess RLE-6TN cell viability. As shown in Figure 3A, exposure to hyperoxia for 72 h significantly reduced cell viability compared with the normoxia control group (Con group) (*P* < 0.05), confirming that hyperoxia induces alveolar epithelial cell injury and death. However, concurrent hydrogen treatment [Hyperoxia + hydrogen-rich medium (HRM) group] markedly improved cell viability, with absorbance values significantly higher than those of the Hyperoxia group (*P* < 0.05), approaching those of the normoxia control. Flow cytometric analysis further demonstrated that hyperoxia increased the apoptosis rate of RLE-6TN cells, whereas HRM treatment significantly reduced apoptosis (Figure 3B). Quantitative PCR revealed that hyperoxia decreased the mRNA expression of AHR and CPEB4, both of which were restored by HRM treatment (Figure 3C). Western blot analysis showed that hyperoxia suppressed AHR phosphorylation, downregulated CPEB4, activated IRE1α, and increased XBP1 expression. Conversely, HRM treatment enhanced AHR phosphorylation, upregulated CPEB4, inhibited IRE1α activation, and reduced XBP1 expression (Figure 3D). To further investigate AHR localization, immunofluorescence analysis revealed that under normoxic conditions, AHR was primarily localized in the nucleus, whereas hyperoxia induced its cytoplasmic translocation (Figure 3E). Notably, after hyperoxia exposure, cytoplasmic AHR levels were markedly elevated, while HRM treatment facilitated a significant redistribution of AHR to the nucleus (Supplementary Figure S2). These findings indicate that HRM promotes AHR nuclear translocation under hyperoxic stress.

### 3.4 AHR promotes the viability of RLE-6TN cells

**Figure 4:**
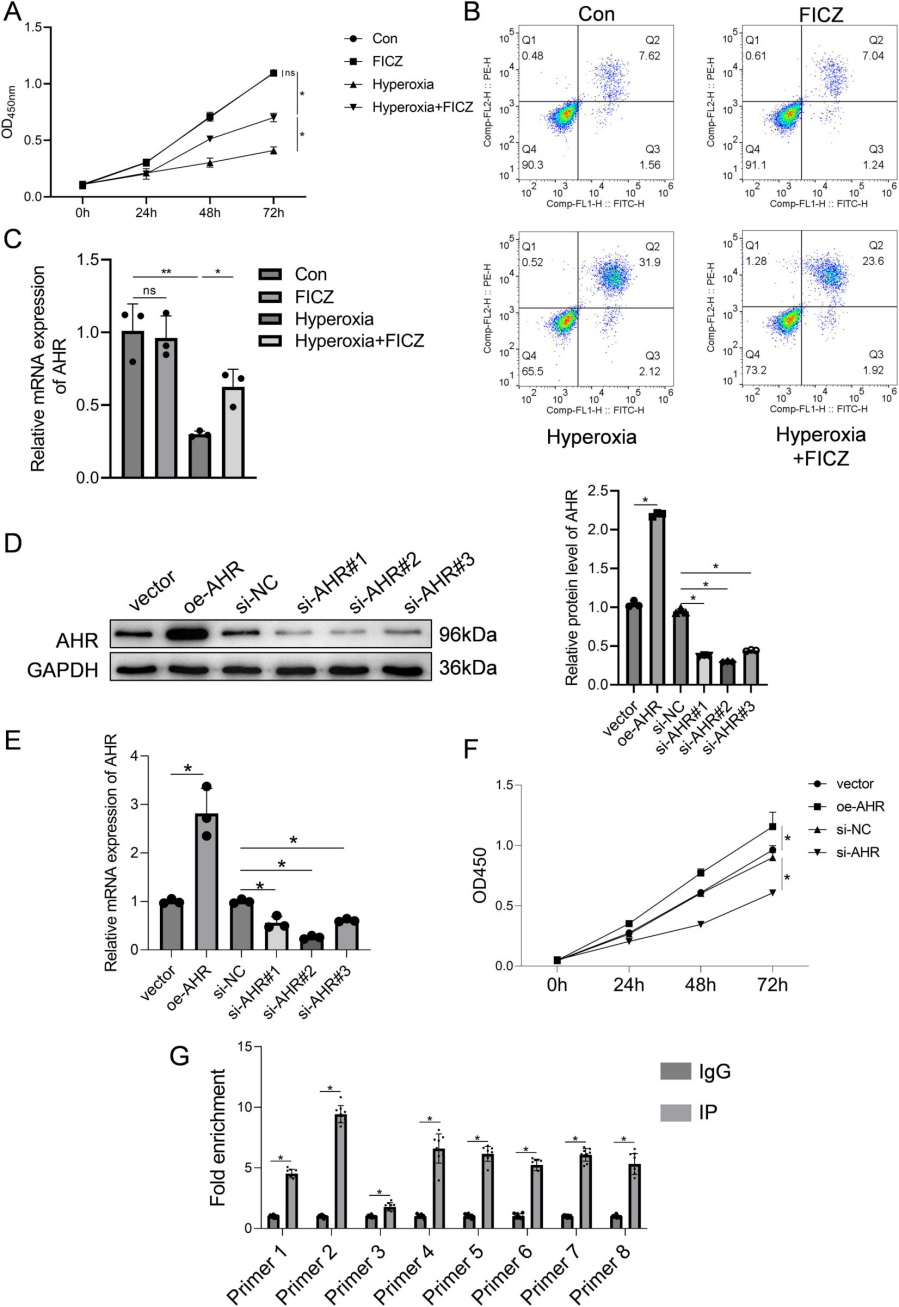
Effect of AHR on RLE-6TN cells in a hyperoxic environment was investigated. **(A)** Cell viability of RLE-6TN cells. **(B)** Apoptosis of RLE-6TN cells. **(C)** mRNA expression levels of AHR in RLE-6TN cells. **(D)** Efficiency of AHR overexpression and knockdown in RLE-6TN cells was verified using western blotting. **(E)** Efficiency of AHR overexpression and knockdown in RLE-6TN cells was verified using qPCR. **(F)** CCK-8 method was employed to assess cell viability in RLE-6TN cells following AHR overexpression and knockdown. **(G)** ChIP-qPCR was utilized to examine the binding of AHR to the CPEB4 promoter (*n* = 9 biological replicates from three independent experiments). Data are presented as mean ± SD, (*n* = 3) for A-F, *P < 0.05.

RLE-6TN cells subjected to hyperoxia were treated with the AHR agonist FICZ. As shown in Figure 4A, hyperoxia markedly reduced cell viability, which was significantly restored following FICZ treatment. Similarly, the apoptosis rate increased under hyperoxia but was significantly reduced after FICZ exposure (Figure 4B). Quantitative PCR showed that hyperoxia suppressed AHR mRNA expression, which was reversed by FICZ (Figure 4C). Western blot analysis demonstrated that hyperoxia inhibited AHR phosphorylation, downregulated CPEB4, activated IRE1α, and elevated XBP1 expression. In contrast, FICZ treatment enhanced AHR phosphorylation, upregulated CPEB4, inhibited IRE1α activation, and decreased XBP1 expression (Supplementary Figure S3).

AHR knockdown and overexpression were validated by qPCR and Western blot. In the overexpression group, AHR protein and mRNA levels were 2.2-fold and 2.8-fold higher, respectively, than in the vector control (Figures 4D,E), confirming successful overexpression. Among the three siRNA constructs tested, si-AHR#2 achieved the most effective knockdown, with AHR protein and mRNA levels reduced to 0.3-fold and 0.26-fold of the si-NC group, respectively; thus, this sequence was selected for subsequent experiments (Figures 4D,E). Functionally, AHR knockdown inhibited RLE-6TN cell viability, whereas AHR overexpression significantly enhanced it (Figure 4F). To determine whether AHR directly binds to the CPEB4 promoter, ChIP-qPCR analysis was performed, confirming a strong AHR binding affinity to the CPEB4 promoter region (Figure 4G).

## 4 Discussion

We utilized a well-established neonatal mouse model of hyperoxia-induced BPD, widely recognized for reproducing the characteristic features of “new BPD” under reproducible experimental conditions (17, 37). The intervention did not affect hepatic or renal function, and all animals demonstrated satisfactory survival. H&E staining revealed simplified alveolar architecture, decreased alveolar counts, enlarged airspaces, and thickened septa in the hyperoxia group compared to controls—confirming successful BPD induction. Given that AHR is highly expressed in oxygen-exchange tissues such as the lung and placenta (38), and that AHR signaling contributes significantly to BPD pathogenesis (26), we investigated its regulatory role. Western blot analysis revealed that hyperoxia inhibited AHR phosphorylation, whereas H₂ treatment restored it and alleviated BPD pathology. Previous studies have shown that AHR activation protects neonatal mouse lungs and human fetal pulmonary microvascular endothelial cells from hyperoxic injury (39) and that pharmacological activation of AHR attenuates oxidative stress in peripheral blood mononuclear cells of premature infants (40).

Our findings further demonstrate that H₂ promotes AHR nuclear translocation, revealing a novel regulatory mechanism. Under physiological conditions, AHR resides primarily in the cytoplasm and becomes transcriptionally active upon nuclear translocation (41). Chromatin immunoprecipitation assays confirmed robust AHR binding to the CPEB4 promoter. Hyperoxia suppressed both AHR phosphorylation and CPEB4 expression, implicating oxidative stress-induced ER stress in translational dysregulation (28). Because CPEB4 controls the translation of mRNAs governing alveolar epithelial cell proliferation and apoptosis, its downregulation disrupts translational homeostasis, promoting apoptosis and alveolar destruction—consistent with TUNEL and H&E findings. H₂ restored CPEB4 expression, stabilized translational balance, and attenuated ER stress by reducing IRE1α phosphorylation and XBP1 expression. IRE1α, a central UPR sensor, becomes activated during ER stress to splice XBP1 mRNA, leading to either adaptive recovery or apoptotic signaling (42). Consistent with prior observations of elevated ER stress in neonatal BPD models (32, 43), we detected increased IRE1α activation and XBP1 levels in both BPD rat lungs and RLE-6TN cells exposed to hyperoxia, underscoring ER stress as a therapeutic target in BPD.

Given antioxidative and cytoprotective properties of hydrogen, we explored its therapeutic efficacy in hyperoxia-induced BPD. Preclinical evidence confirms that 2%–4% H₂ is safe, diffusible, and effective in neutralizing reactive oxygen species (ROS) without impairing oxygenation (44). We employed a custom gas delivery system generating a 2% H₂–O₂ mixture—optimal for efficacy and safety, as concentrations >4% are flammable and <1% ineffective (45). Based on Henry's law, inhaled 2% H₂ achieves plasma concentrations of ∼0.4–0.6 mm (46), while methylene blue titration confirmed that HRM (0.6 mm) maintains stable H₂ levels for 24 h (47). Our results show that H₂ ameliorates hyperoxia-induced BPD by modulating the AHR–CPEB4–ER stress axis and enhancing surfactant synthesis. SP-A and SP-B, crucial for reducing alveolar surface tension (48) and maintaining lung elasticity, were markedly decreased in BPD rats but restored following H₂ treatment. In RLE-6TN cells, H₂ enhanced AHR phosphorylation and nuclear translocation, upregulated CPEB4 via direct promoter interaction, improved cell viability, and reduced apoptosis. These effects paralleled those of the AHR agonist FICZ and were abolished by AHR knockdown, confirming AHR's pivotal role in H₂-mediated cytoprotection under hyperoxic stress.

In summary, our study identifies a novel mechanism wherein H₂ activates AHR to upregulate CPEB4, thereby suppressing ER stress, enhancing surfactant protein synthesis, and improving pulmonary outcomes in neonatal BPD. Nevertheless, several limitations warrant consideration. RLE-6TN cells, derived from adult rat alveolar epithelium, may not fully recapitulate the immature phenotype of neonatal lungs. Future research will employ primary alveolar epithelial cells isolated from postnatal day 1–3 rats to better reflect developmental biology. In *vivo*, longer observation periods are needed to evaluate functional and structural recovery, including body weight, tidal volume, respiratory rate, airway resistance, and histopathological parameters such as alveolar simplification, vascular remodeling, and inflammation. To define precise dose–response relationships, H₂ levels in biological matrices will be quantified using liquid chromatography–mass spectrometry (LC–MS). Additionally, as paraffin embedding may cause unpredictable tissue shrinkage (49), complementary morphometric methods should be incorporated for accuracy. Finally, testing whether AHR antagonists abolish H₂'s protective effects will be critical to confirm causality. The observed discrepancy between *in vivo* and *in vitro* apoptotic responses—potentially due to compensatory antioxidant mechanisms present only *in vivo*—underscores the need for physiologically relevant platforms such as lung organoids.

## 5 Conclusion

Collectively, these findings suggest that H₂ alleviates ER stress and reduces apoptosis through AHR–CPEB4 activation, representing a promising therapeutic avenue for hyperoxia-induced BPD.

## Data Availability

The original contributions presented in the study are included in the article/Supplementary Material, further inquiries can be directed to the corresponding authors.
