# Supplementary material for: Hydrogen regulates the aryl hydrocarbon receptor, improving bronchopulmonary dysplasia in neonatal rats and RLE-6TN cells exposed to hyperoxia
Source: Front Pediatr. 2025 Nov 17;13:1662922. doi: 10.3389/fped.2025.1662922 (PMC12665534; doi:10.3389/fped.2025.1662922)

Supplementary Material

**Supplementary Data 1.** **Synthesis of Overexpression Plasmid Constructs**

>Rat-AHR

GAATTCGCCACCatgagcagcggcgccaacatcacctatgccagccgcaagcggcgcaagccggtgcagaaaacagtaaagcccgtccctgctgaaggaattaagtcgaacccttctaaacgacacagagaccggctgaacacagagttagaccgcctggctagcctgctgcccttcccacaagatgttattagtaagctggacaaactctccgttctaaggctcagcgtcagctacctgagggccaagagcttctttgatgttgcattaaaatccaccccggctgacagaagtagaggccaggaccagtgtagagcacaagtcagagactggcaggacttgcaagaaggagagttcttgttacaggcgctgaatggctttgttctggttgtcacggcagatgccttggtcttctatgcgtcttccactatccaagattacctgggctttcagcaatctgatgtcatacatcagagcgtgtatgagcttatccatacagaagaccgagctgagttccagcgccagcttcactgggctctaaacccctcacagtgcacagactctgcacaaggagtagacgagactcatggcctcccacagccagcggtctactacacgccagaccagcttcctccagagaataccgctttcatggagaggtgcttcagatgccggctgaggtgcctgctggataattcatctggtttcctggcaatgaatttccaagggaggttaaagtatcttcatggacagaacaagaaagggaaagacggagcgctactccctccacagttggctttgtttgcaatagctactccacttcagccaccgtccatcctggaaattcgaaccaaaaacttcatcttcaggaccaaacacaaactggacttcacacctattggctgtgatgccaaagggcagcttattctgggctacacagaagtagagctgtgcaacaaaggatcgggatatcagtttatccacgccgctgacatgcttcactgcgcagaatcccacatccgcatgattaagactggagaaagtggcatgacagttttccggcttcttgcaaaacacagtcgatggaggtgggtccagtccaatgcacgcttgatttacagaaatggaagaccagattacatcatcgcaactcagagaccgctaacggatgaagaaggacgcgaacatttacagaagagaagtatgacactgccattcatgtttgccactggaaaggctgtactgtacgagatctccagccctttctctcccataatggatcccttgccaatacgcaccaaaagcaacactagtaggaaagactgggctccccagtcaaccccgagtaaggattctttccaccccaattcccttatgagtgccttgatccaacaggacgagtccatctatctctgtcctccttcgagccccgcaccattagacagccattttctcatggactccatgagtgagtgcggcagttggcaaggcagctttgcagccgcaagcaatgaagctctgctgaaacacgaggaaatcagacacactcaggacgtgaaccttacactctctggaggcccctcggagctcttcccagataataaaaataatgacttgtatagcatcatgagaaacctagggatcgatttcgaagacatcagaagcatgcagaatgaggagttcttccgaaccgactcctccggtgaggttgacttcaaagacatcgacataacagacgaaatcctgacgtacgtgcaggattctctgaacaattcaactctgctgaattcagcttgccagcaacagcctgtgagccagcacctaagctgcatgctgcaggagcgcctgcagctggagcaacaacagcagcttcagcagcagcaccccactcagacactggagccccagcgccagttgtgtcaggtggaggtcccccagcacgagctgggtcagaaaacgaagcacatgcaagtcaatggcatgttcgccagttggaaccctgcccctcccgtgtctttcagctgtcctcagcaggaacgaaagcactatagcctcttctccggcttacaggggactgcacaggagtttccctacaagtcagaggtggacagtatgccttacacacagaactttgctccctgcaaccagtcactgctaccagaacattccaagggtacacagttggacttccctggaagggattttgaacgatccctgcaccctaacgcttctaatttagaagactttgtcagttgtttacaagttcctgaaaaccaaagacacgggataaactcacagtcagccatggtcagtcctcaggcgtactacgctggggccatgtccatgtaccagtgccaggcagggcctcagcacacccctgtggaccagatgcagtacagccctgagattccaggctcccaggcgttcctaagcaagtttcagagtccgagcattttaaatgaagcctactcggcagacttgagcagcattggccaccttcagactgctgctcacctccctcgcctggcagaagcccagcctcttcctgatatcacacccagcggattcctgtagGGATCC


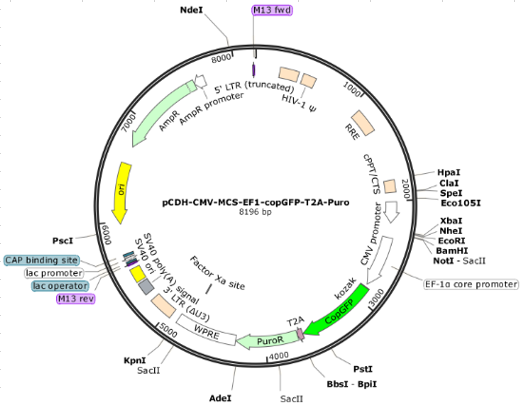


**Supplementary Data 2.** **siRNA Sequence**

si-rat-Ahr-1：GAGAGUUCUUGUUACAGGCGCUGAA and UUCAGCGCCUGUAACAAGAACUCUC

si-rat-Ahr-2：GGGCUUUCAGCAAUCUGAUGUCAUA and UAUGACAUCAGAUUGCUGAAAGCCC

si-rat-Ahr-3：CAGAUAAUAAAAAUAAUGACU和UCAUUAUUUUUAUUAUCUGGG

si-rat-control：GAGCUUGUUAUUGACGCGUCAGGAA and UUCCUGACGCGUCAAUAACAAGCUC

**Supplementary Data 3.** **Concentration of plasmids**

**si-RNA**

liquid A: 100 μL Opti-MEM + 100 pmol siRNA

liquid B: 100 μL Opti-MEM + 5 μL RNAiMAX

**oe-RNA**

4-5k ng/μl

**Supplementary Data 4.** **Primer Sequences Required for ChIP-qPCR**

Primer 1 (TSS-606~-420)：GGTGATAGAATTATAAAAGGATAATTTG and CTAAAATCCCAACATCTATATCCCC

Primer 2 (TSS-519~-62)：GTGTTGTGTTGTAGTTTTTGATTGG and AAACATAACTTCAAATAACCCCTTC

Primer 3 (TSS-519~-366)：GTGTTGTGTTGTAGTTTTTGATTGG and AACACCAAAACTCTAATTCCTAAAA

Primer 4 (TSS-517~-61)：GTTGTGTTGTAGTTTTTGATTGGG and AAAACATAACTTCAAATAACCCCTTC

Primer 5 (TSS-517~-62)：GTTGTGTTGTAGTTTTTGATTGGG and AAACATAACTTCAAATAACCCCTTC

Primer 6 (TSS-445~-61)：GGGGATATAGATGTTGGGATTTTAG and AAAACATAACTTCAAATAACCCCTTC

Primer 7(TSS-445~-62)：GGGGATATAGATGTTGGGATTTTAG and AAACATAACTTCAAATAACCCCTTC

Primer 8(TSS-445~-61)：GGGGATATAGATGTTGGGATTTTAG and AAAACATAACTTCAAATAACCCCTT

**Supplementary Data 5.** **CPEB4 promoter region**


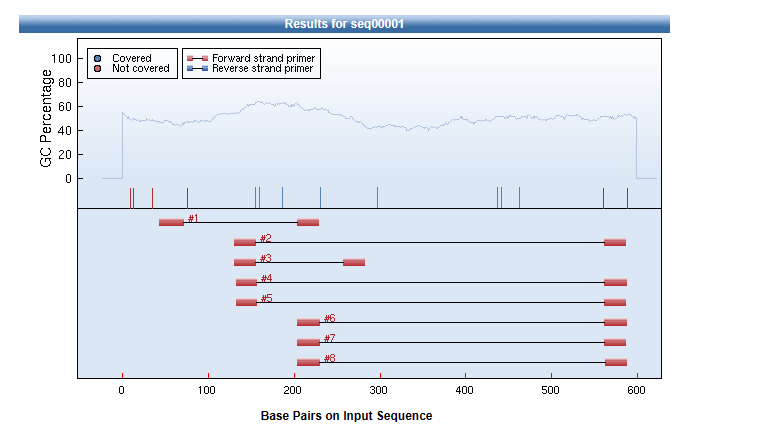

Supplement: Supplementary file 1 [file Datasheet1.docx]
