# Supplementary material for: Hydrogen regulates the aryl hydrocarbon receptor, improving bronchopulmonary dysplasia in neonatal rats and RLE-6TN cells exposed to hyperoxia
Source: Front Pediatr. 2025 Nov 17;13:1662922. doi: 10.3389/fped.2025.1662922 (PMC12665534; doi:10.3389/fped.2025.1662922)
Supplement: Supplementary file 2 [file Datasheet2.docx]

Supplementary Material


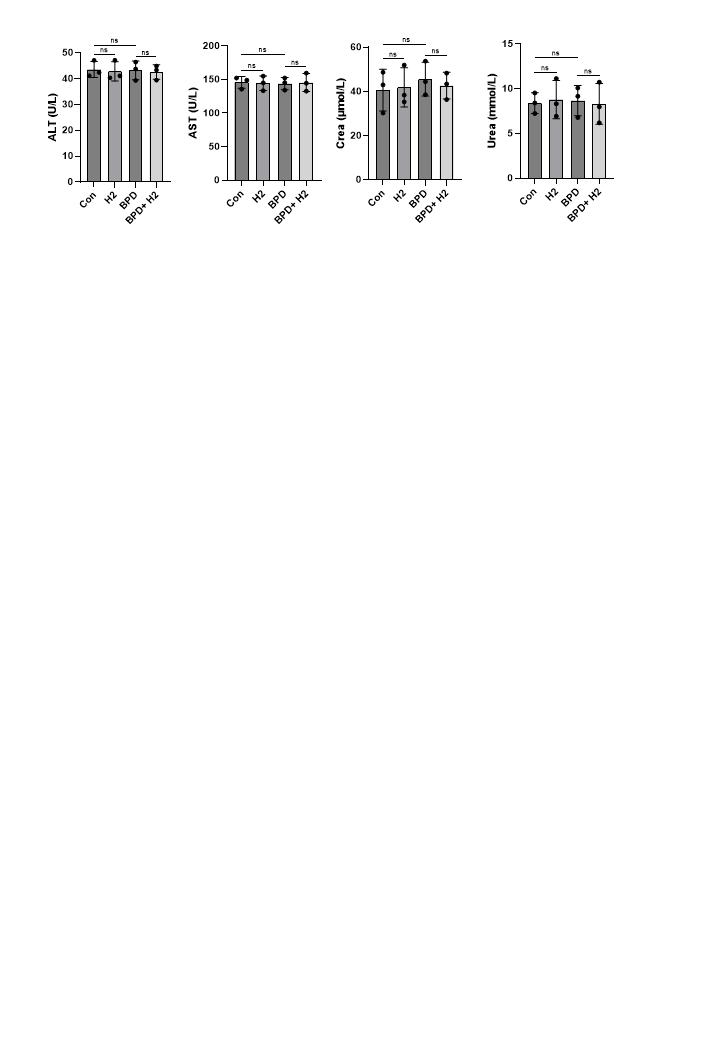


**Supplementary Figure 1.** Serum biochemical indicators of rats in different treatment groups: ALT(U/L)、AST(U/L)、Crea(μmol/L)、Urea(mmol/L), Data are presented as mean ± SD. n = 3, *P < 0.05.


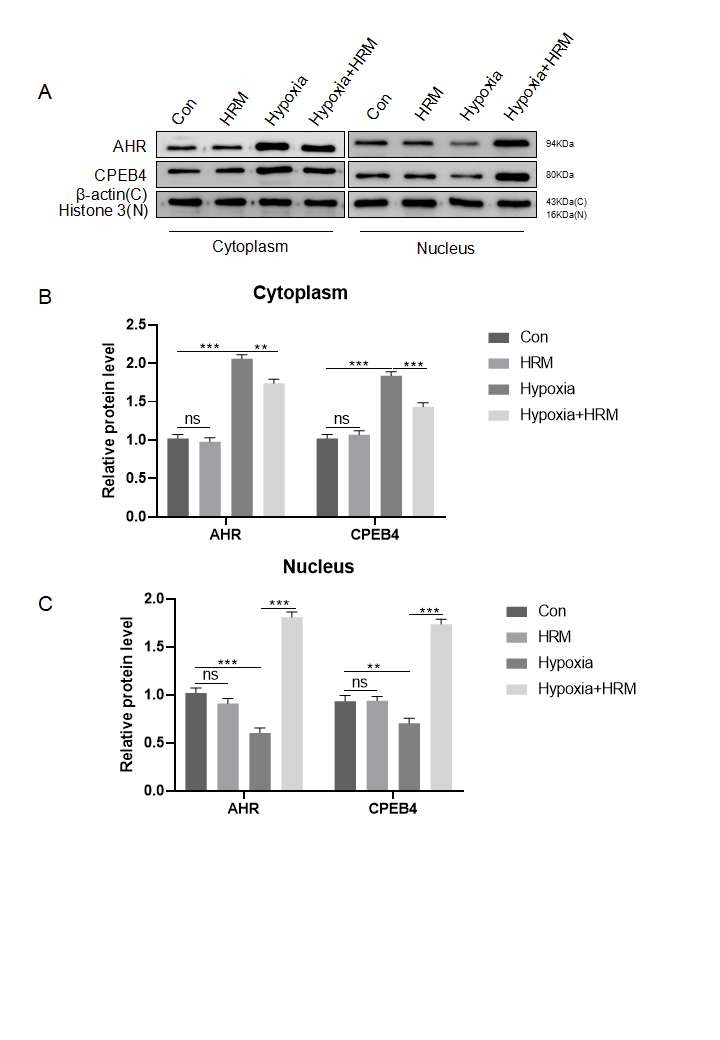


**Supplementary Figure 2.** H_2_ treatment led to the nuclear translocation of AhR in MC38 cells. (A-C) The nuclear and cytoplasmic components were separated. The localization of AhR was detected by Western blot analysis (n = 3). The Western blot plot showed that the nuclear-cytoplasmic ratio of AhR protein was quantified by colorimetry and standardized with the corresponding internal reference protein. Data are presented as mean ± SD. n = 3, *P < 0.05.


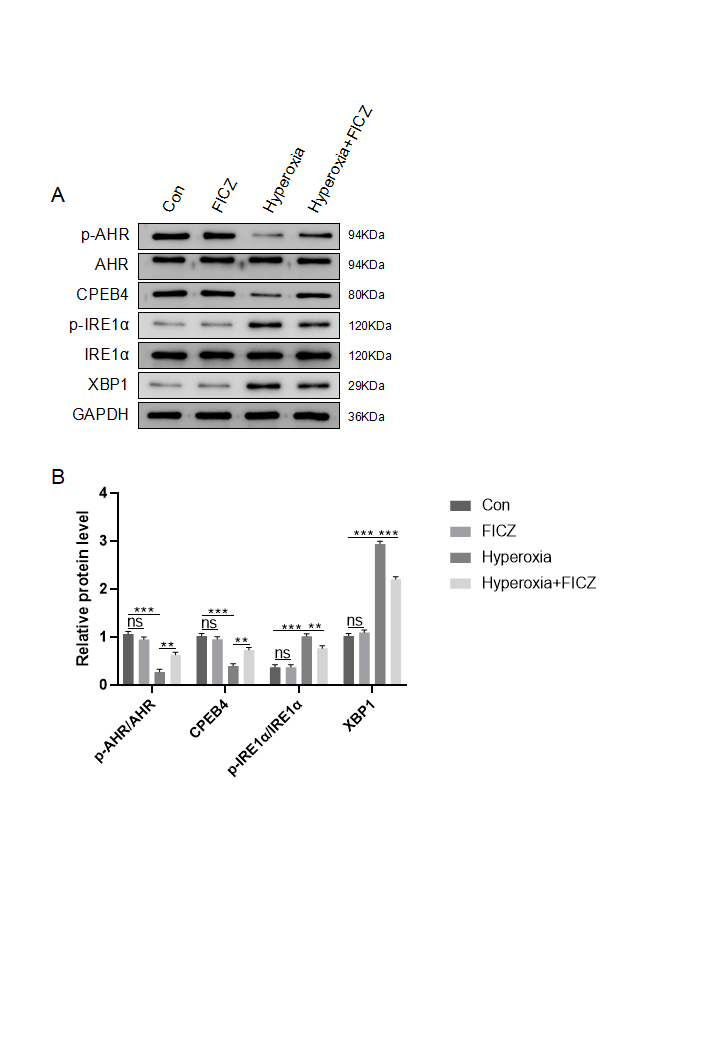
 **Supplementary Figure 3.** （A-B）WB analysis following FICZ treatment was conducted to assess the expression of p-AHR, AHR, CPEB4, p-IRE1α, IRE1α, and XBP1 in RLE-6TN cells, followed by quantitative analysis of the protein bands.
